# Healing and Spontaneous Realignment of Displaced Roots With Periapical Granuloma After Microsurgical Endodontic Treatment (Three Years’ Follow-up): A Case Report

**DOI:** 10.7759/cureus.52020

**Published:** 2024-01-10

**Authors:** Omar Alzahrani, Hisham Komo, Mohammed Howait

**Affiliations:** 1 Department of Advanced General Dentistry, Faculty of Dentistry, King Abdulaziz University Dental Hospital, Jeddah, SAU; 2 Department of Oral and Maxillofacial Surgery, Faculty of Dentistry, King Abdulaziz University Dental Hospital, Jeddah, SAU; 3 Department of Endodontics, Faculty of Dentistry, King Abdulaziz University, Jeddah, SAU

**Keywords:** root canal treatment, mineral trioxide aggregate, dental healing, apicoectomy, apical microsurgery

## Abstract

Endodontic therapy aims to treat or prevent apical periodontitis, a condition characterized by inflammation of the periapical tissues at the apex of the tooth root. This case study demonstrates the successful nonsurgical and surgical management of a large periapical lesion involving the lower central incisors with root displacement induced by a periapical granuloma. A patient was referred from the maxillofacial department for endodontic treatment due to persistent pain and swelling in the lower anterior region started two months ago. Upon arrival, a clinical examination and radiographic assessment were performed using cone-beam computed tomography (CBCT). The CBCT scan revealed a significant radiolucent area measuring (10x8) mm extending from the lower left lateral incisor to the right central incisor. The lower left central incisor was necrotic and tender to palpation and percussion. A nonsurgical root canal was performed followed by an apicectomy using mineral trioxide aggregate (MTA) to facilitate healing of the periapical lesion. Histopathological examination of the lesion confirmed the diagnosis of periapical granuloma. At follow-up 1, 2, and 3 years' visits, the periodontal assessment was performed and found to be free of pain upon percussion or palpation. No other clinical or radiological signs or symptoms were identified except for a small radiolucent area mesially adjacent to the root of the lower left central incisor. The development of materials such as MTA has significantly improved the prognosis of cases with large periapical lesions. In this case, healing and spontaneous realignment of the root were observed after three years.

## Introduction

According to the guidelines set by the American Association of Endodontists, the fundamental goal of endodontic treatment is to achieve a cure or to prevent the occurrence of apical periodontitis. They are a prevalent complication of untreated dental caries and can also arise from trauma or other tooth injuries [1]. The primary cause of periapical lesions is infection. Root canal treatment is the primary therapy for periapical lesions. The objective of root canal treatment is to eliminate infected pulp tissue and bacteria from the tooth and to seal the root canals to prevent reinfection [2]. Numerous factors can potentially lead to the failure of non-surgical root canal treatment, with persistent infection being a prominent contributing factor [3]. However, tooth extraction should not be justified by endodontic failure alone. In cases with recurrent or persistent infection, there are alternative treatments, such as non-surgical and surgical approaches [4]. A radiographic lesion with a diameter exceeding 8 or 10 mm is considered significant in size [5]. If a periapical cyst is suspected, endodontic surgery is necessary to remove the cyst, and a histological biopsy should be performed to confirm the diagnosis [6]. The efficacy of surgical root canal treatment is reported to range from 76% to 86% using strict criteria [7]. Additionally, various materials are currently used in the field of endodontics.

The successful treatment of periapical cysts and other endodontic conditions relies heavily on the choice of filling material used during retrograde filling procedures. Mineral trioxide aggregate (MTA) has emerged as the preferred material due to its exceptional sealing ability, biocompatibility, and regenerative potential for tissue formation [8,9]. MTA has become the preferred material for retrograde filling due to its exceptional sealing ability, biocompatibility, and regenerative potential for tissue formation. Compared to other retrograde filling materials, such as amalgam or glass ionomer, which were used in the past, MTA has a significantly higher success rate. MTA has a significant advantage in its ability to stimulate cementum-like tissues, with a reported success rate of 94% when used as an apical plug [10].

Furthermore, periapical granulomas comprise 50% of all periapical lesions. They are chronic inflammatory lesions that develop around the apex of a tooth root in response to persistent dental infection, and they can exhibit two distinct histological forms: epithelialized and non-epithelialized. Epithelialized periapical granulomas, accounting for 45% of all granulomas, are characterized by the presence of epithelial lining within the lesion. This epithelial lining can arise from remnants of Hertwig's epithelial root sheath (HERS) or from the periodontal epithelium. The presence of epithelium within the granuloma may be associated with a higher risk of periapical cyst formation. And non-epithelialized periapical granulomas, comprising 55% of all granulomas, lack epithelial lining and are instead composed of a dense infiltrate of chronic inflammatory cells, primarily lymphocytes and plasma cells. These granulomas are considered to be less likely to progress to periapical cysts compared to their epithelialized counterparts [11]. Surprisingly, despite reports of spontaneous root realignment following surgical treatment, these lesions are not reported in the literature as a cause of root displacement. The incidence of root displacement is less frequent with granuloma than with periapical cyst [12]. Therefore, this case study describes the nonsurgical and surgical (endodontic) treatment of a large periapical lesion with root displacement.

## Case presentation

The maxillofacial department referred a 29-year-old male patient for root canal treatment prior to surgical excision of a large radiolucency around the root apices of the mandibular central incisors teeth. The primary presenting symptoms were localized to the mandibular anterior region extending from the mandibular left lateral incisor to the mandibular right central incisor and it was soft and non-fluctuant with a slight tenderness to palpation, and dull continuous pain associated with the affected area without any aggravating factors that started two months ago. The patient has no known medical history and dental history revealed a trauma 14 years ago (fell on his chin). Upon oral examination, vestibular swelling to his lower anterior teeth was observed. Tooth #31 was non-responsive to cold and electric pulp tests, and pain to percussion leading to a diagnosis of necrosis with symptomatic apical periodontitis. Responses of teeth #32 and #41 to sensibility testing (electric pulp and thermal tests) were found normal.

**Figure 1:**
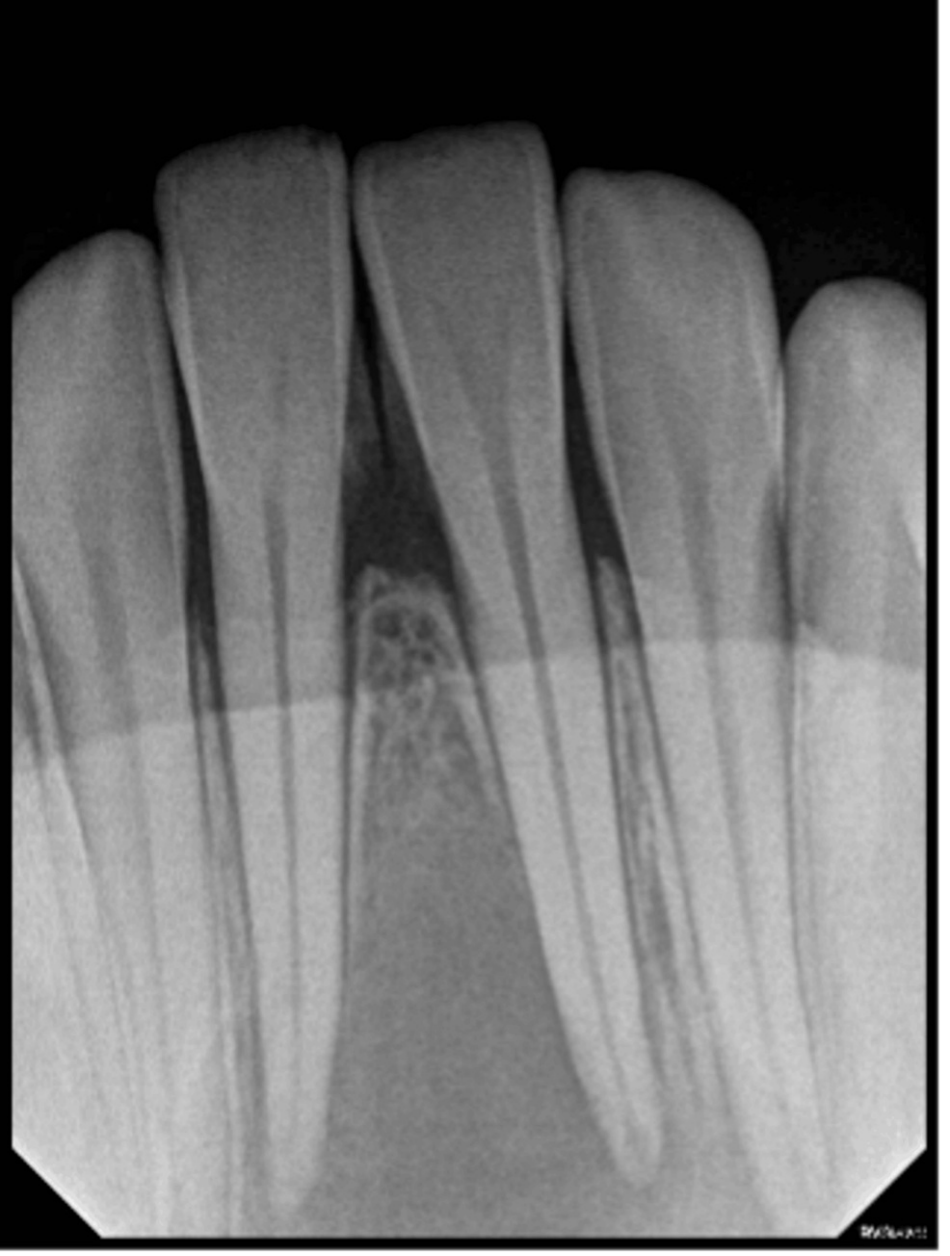
Intraoral preoperative periapical radiograph A large radiolucency was observed in the radiographic examination, extending from the left lateral incisor to the right lateral incisor, showing at the root apex and displacing the roots of both mandibular incisors.

**Figure 2:**
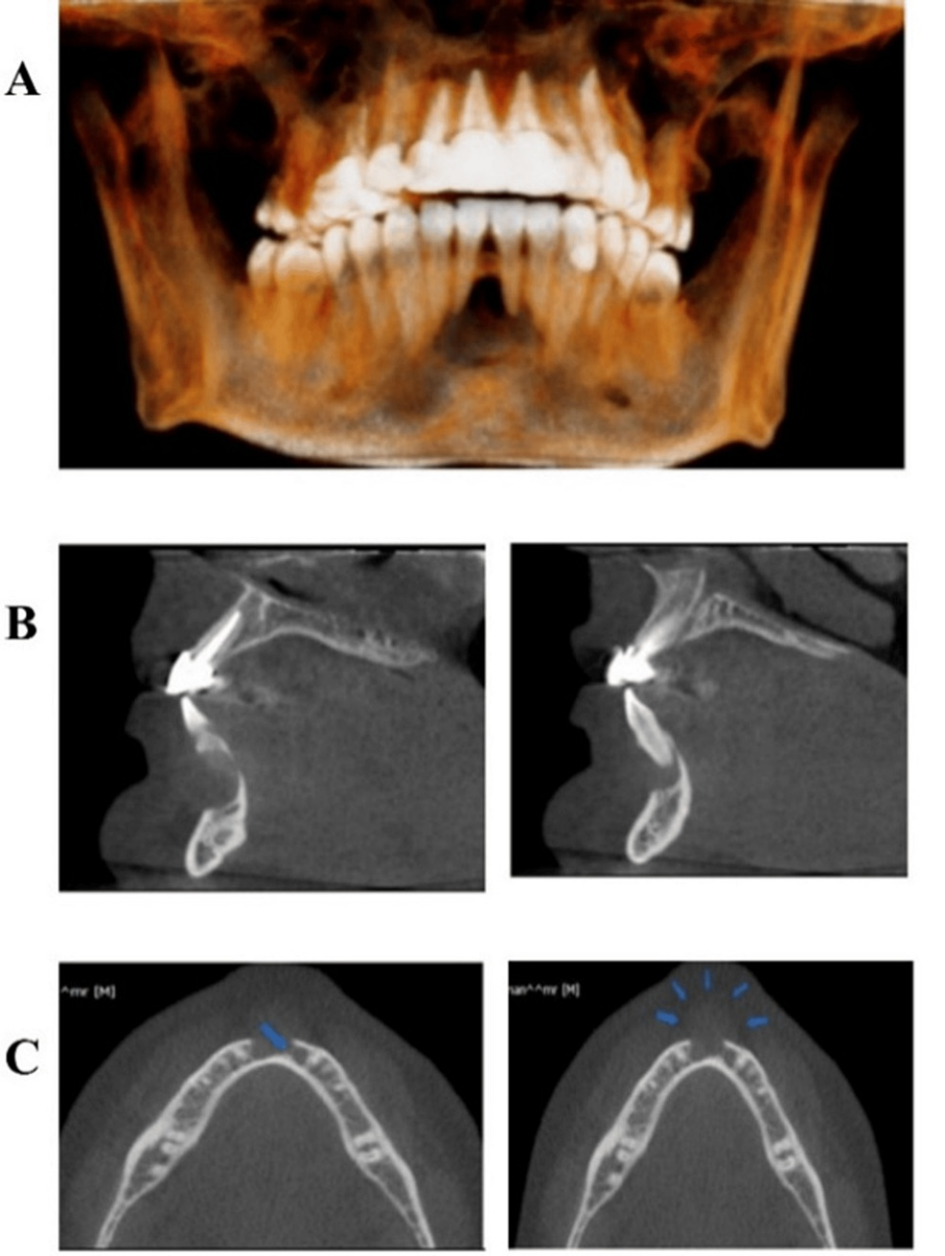
(A) Cone-beam computed tomography (CBCT) (Reconstructed). (B) Lateral view. (C) Axial view The imaging procedures detected the presence of a well-demarcated, non-corticated, pear-shaped radiolucent lesion in the anterior region of the mandible, extending from tooth #33 to tooth #43 (Figure 2A). The lesion caused the slight separation of roots of the lower central incisors, breached the buccal plate of bone, and thinned and eroded the lingual plate of bone (Figure 2B). There is evidence of faint septation within the lesion (Figure 2C). The soft tissue component of the lesion extended to the overlying labial soft tissues (Figure 2C). Based on the observed location and behavioral patterns, central giant cell granuloma is the most likely diagnosis. However, inflammatory cysts and keratocysts cannot be excluded.

**Figure 3:**
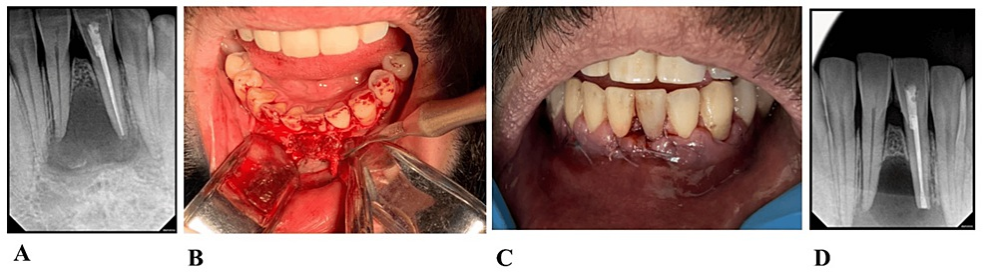
(A) Postoperative radiograph after non-surgical root canal treatment. (B) Intraoperative photograph. The lesion after flap reflection. (C) Suture removal after 5 days. (D) Postoperative radiograph after root canal surgical treatment of the lower left central incisor. The dental procedure involved isolating the tooth with a rubber dam, followed by canal access, cleaning, and shaping to the entire working length. Subsequently, a hydraulic condensation technique was used to obturate the canal using a bio-ceramic sealer (Endosequence BC Sealer)(Figure 3A). The tooth was scheduled for apicoectomy and surgical excision of the lesion (Figure 3B). During the second scheduled visit, microsurgical root canal treatment was performed using ProRoot MTA. The lesion was enucleated and subsequently sent for histopathological examination, which revealed a periapical granuloma.

**Figure 4:**
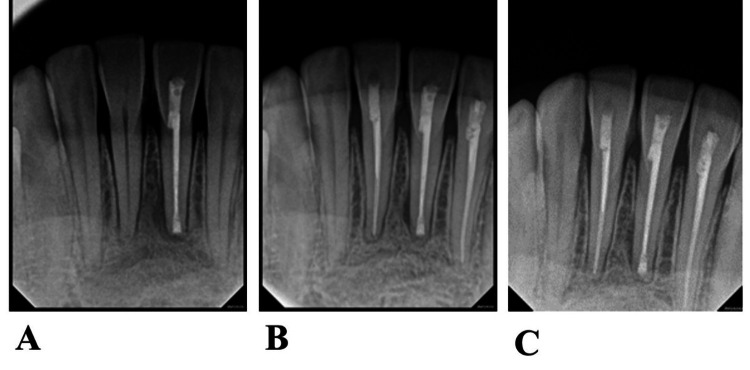
(A) Periapical radiograph after 12, (B) 24 and (C) 36 months’ follow-up A subsequent examination was conducted after 12, 24, and 36 months (follow-up) (Figure 4A-4C), which revealed a decrease in the size of the lesion. Additionally, no pain was reported during percussion and palpation. Furthermore, there was no observation of vestibular swelling.

The clinical assessment was conducted, encompassing an evaluation of the soft tissues, tooth mobility, and any signs of inflammation or discomfort. The study documented the patient's self-reported feedback regarding symptoms or changes in oral health, as well as histopathological evaluation. Radiographic images, including periapical or cone-beam computed tomography (CBCT) scans, were acquired during every subsequent examination (Figures 1-4). One endodontist and one maxillofacial surgeon evaluated the images to ascertain any changes in root position, periapical healing, and bone regeneration. Periapical pathology indicators were documented, such as periapical radiolucency or bone loss.

The patient was given written informed consent, in accordance with the Helsinki Declaration on the Ethical Principles for Medical Research Involving Human Subjects for the publication of his case report. The procedure was conducted by an endodontic resident utilizing a surgical operating microscope. Anesthesia was given using two carpules of 4% articaine with 1:100,000 epi as buccal infiltration. The rubber dam was applied and access cavity was made using a size 3 mm in diameter round diamond bur (Dentsply Maillefer, Ballaigues, Switzerland) at high-speed underwater spray; determining the working length using a DentaPort ZX apex locator (J. Morita Mfg. Corp., Kyoto, Japan) Reciproc® blue (R25/0.08 mm) (VDW GmbH, Munich, Germany) was used for cleaning and shaping to full working length, and irrigation using 15 mL of 3% NaOCl using a 30-gauge side vented irrigating needle (Max-I-Probe, Dentsply Maillefer, Ballaigues, Switzerland). The canal was dried using an absorbent paper point size 25 red color code (Meta Dental Corp., New York, USA), and Cold single-cone technique (hydraulic condensation technique) with (#25/0.08) matching gutta-percha cones and bioceramic sealer were used as an obturation method. Total Fill® bio-ceramic sealer (TotalFill BC Sealer) (FKG Dentaire, La Chaux-de-Fonds, Switzerland) was applied. One week later the surgery was scheduled and anesthesia was given using two carpules of 4% articaine with 1:100,000 epi as buccal infiltration and 1 carpule of lidocaine 1:50,000 epi distributed along the mucogingival line from tooth #33-42. A triangular full mucoperiosteal flap was raised with a mesial vertical releasing incision. Lesion enucleation was carried out followed by root resectioning of 3 mm using Lindemann bur and retropreparation using ultrasonic and retrograde filling using MTA. Suturing was done using 5-0 nylon and removed five days later.

The patient received postoperative instructions and oral hygiene protocols. Periodic follow-up appointments were arranged for clinical and radiographic assessments at 1, 2, and 3-year intervals which show healing of the periapical lesion except slight widening on the mesial side of the root of tooth number #41. The research complied with ethical guidelines and the principles stated in the Declaration of Helsinki, adopted in 1964.

Histopathological samples sent for microscopic examination consisted of three fixed pieces of soft tissue, tan-black in color, and measuring aggregate of 2.5×1.3×1.0 cm. The largest was bisected and revealed multiple fragments of granulation tissue infiltrated by a mixed chronic inflammatory cell infiltrate, with hemorrhagic bleeding and trabeculae of vital bone. The diagnosis was periapical granuloma.

## Discussion

Periapical lesions in teeth are caused by the infiltration of bacteria into the root canal system, which can persist if the canal is not cleaned or filled properly. Root displacement is an infrequent complication of these lesions [12]. However, root displacement was observed in the present case report, and subsequent healing resulted in continuous realignment.

Apicoectomy is a viable treatment option for cases of persistent infection. It has a high success rate and is more predictable than other treatments in eliminating persistent infection. Apical surgery has a success rate of 75-90% within six months [5]. Despite its high success rate, apicoectomy is not without drawbacks. Potential risks include root canal failure, tooth structure loss, and postoperative complications. Apicoectomy is typically considered when conventional endodontic therapy fails, such as in cases of complex root canal anatomy or apical pathology with inadequate access. Contraindications include severe periodontal disease, extensive root resorption, inadequate bone support, anatomically inaccessible roots, and uncontrolled medical conditions. Alternative treatment options may be considered in these cases [13,14].

Mineral trioxide aggregate (MTA) has emerged as a versatile and effective root-end filling material, particularly in cases of complex root canal anatomy or persistent apical infection. Its remarkable sealing ability, stemming from its hydraulic setting properties and ability to adhere to dentin and bone, contributes to its success in preventing reinfection and promoting healing. MTA's unique physicochemical properties, including its biocompatibility, non-resorbability, and ability to induce cementogenesis, further enhance its suitability for apical repair procedures. Clinical applications of MTA encompass a wide range of endodontic treatments, including apexification, apexogenesis, repair of root perforations, root-end fillings, and vital pulp therapy. Its versatility and effectiveness have made MTA an indispensable material in the armamentarium of modern endodontics [15,16].

Teeth with large periapical lesions measuring 8-10 mm should be excised through surgical intervention, as most of these lesions are cystic and can only be completely healed through surgery [5]. Numerous studies have demonstrated the efficacy of surgical intervention for large periapical lesions. A systematic review by Baseri et al. concluded that apical surgery achieved a significantly higher rate of complete healing (92.8%) compared to non-surgical treatment (74.1%) for large periapical lesions [17]. Similarly, a meta-analysis by Ng et al. reported a pooled success rate of 90.3% for apical surgery in treating large periapical lesions, significantly higher than the success rate of 74.3% for conventional RCT [18].

While Ghorbanzadeh et al. reported a case with similar presenting symptoms that achieved complete resolution with only non-surgical management, the patient in the present case exhibited a distinct clinical course. Despite non-surgical interventions, the pain and swelling persisted, highlighting the need for surgical intervention [19]. A comparable study by Oztan described a case involving all lower incisors affected by a single traumatic incident. Notably, three of the involved teeth exhibited non-responsiveness to electric pulp testing, necessitating non-surgical management. This approach resulted in periapical healing within three months. In contrast, the present case report presents a scenario with only one tooth displaying negative electric pulp test results at the initial examination. Despite non-surgical intervention, this case yielded no observable signs of healing or symptom resolution [20]. In the present study, the lesion was large and was histopathologically verified as a periapical granuloma. However, the teeth spontaneously realigned as the lesion healed without orthodontic intervention.

## Conclusions

The present case study successfully demonstrates the combined efficacy of nonsurgical and surgical endodontic approaches in managing a periapical granuloma affecting the lower central incisors and facilitating spontaneous root realignment after displacement.
